# Prospective 5 year outcomes of different implant designs and surgical techniques in 68 patients with bone anchored hearing implants

**DOI:** 10.1111/coa.13974

**Published:** 2022-09-17

**Authors:** Maarten A. Vijverberg, Coosje J. I. Caspers, Ivo J. Kruyt, Emmanuel A. M. Mylanus, Myrthe K. S. Hol

**Affiliations:** ^1^ Donders Center for Neurosciences, Department of Otorhinolaryngology Radboud University Medical Center Nijmegen Netherlands; ^2^ Department of Otorhinolaryngology/Head and Neck Surgery University Medical Center Groningen, University of Groningen, Groningen, Netherlands; Research School of Behavioral and Cognitive Neurosciences, Graduate School of Medical Sciences, University of Groningen Groningen Netherlands

**Keywords:** bone conduction, bone‐anchored hearing, hearing loss, implant stability quotient, tissue preservation, wide diameter implant

**Key Points**

1. Both the 4.5‐mm‐wide implant and the 3.75‐mm‐wide implant have excellent survival rates at 5‐year follow‐up.
2. Further research supporting larger sample sizes should be conducted to detect a difference in survival rate between the 4.5‐mm‐wide implant and the 3.75‐mm‐wide implant.
3. At 5 years, no significant differences in adverse skin reactions nor skin sensitivity were observed between the linear incision technique with soft tissue reduction (LIT‐TR) and the linear incision technique with soft tissue preservation (LIT‐TP).
4. Because of the shorter surgery time, less invasive character and comparable clinical outcomes, the LIT‐TP is preferred over the LIT‐TR.
5. In the long term, the use of both a 3.75‐mm‐wide and a 4.5‐mm‐wide percutaneous implant and the tissue preservation procedure leads to high implant survival with a low percentage of adverse skin reactions.

## 1 INTRODUCTION

The 4.5‐mm‐wide implant introduced in 2011 had larger bone‐to‐implant contact surface compared with the 3.75‐mm‐wide implants, and has been shown superior in terms of higher implant stability quotient (ISQ).^1^, ^2^ Although implant survival of the 4.5‐mm‐wide implant is high, differences in survival have not been found when compared with 3.75‐mm‐wide implant. Striving to decrease adverse skin reactions and improve skin sensitivity, the linear incision technique with soft tissue reduction (LIT‐TR) was modified into a linear incision technique with soft tissue preservation (LIT‐TP), demonstrating more favourable results regarding skin sensitivity and cosmetic outcomes.^3^, ^4^, ^5^ Although short‐term outcomes are favourable, limited data is available on long‐term outcomes of the 4.5‐mm‐wide implant and concomitant surgical techniques.^6^, ^7^, ^8^

This study compares 5‐year clinical outcomes of a 4.5‐mm‐wide implant and a 3.75‐mm‐wide implant. Furthermore, 5‐year outcomes of the LIT‐TP and the LIT‐TR are compared.

## 2 MATERIALS AND METHODS

### 2.1 Study design

This study was set up as a prospective single follow‐up visit, 5 years after Bone‐Anchored Hearing Implant (BAHI) implantation for patients who had completed the follow‐up of two previously published studies, conducted at our tertiary referral center. These studies investigated 6‐month and 3‐year clinical outcomes of a 4.5‐mm‐wide implant versus a 3.75‐mm implant (study A),^7^, ^8^ and the LIT‐TP versus the LIT‐TR (study B).^5^, ^6^ In‐ and exclusion criteria for these two studies were identical and have been previously described.^5^, ^7^

#### 2.1.1 Study A: 4.5‐mm‐wide implant versus 3.75‐mm‐wide implant

Study A compared clinical outcomes of the 4.5‐mm‐wide implant with the 3.75‐mm‐wide implant.^7^, ^8^ Fifty‐seven patients (60 implants) were included and randomised into a test‐ (37 patients with 40 implants) and control group (20 patients with 20 implants). The test group was implanted with the Wide Ponto implant (diameter 4.5 mm, length 4 mm, Oticon Medical AB, Askim, Sweden). The control group received the previous generation Ponto implant (diameter 3.75 mm, length 4 mm, Oticon Medical AB, Askim, Sweden). A 6‐mm abutment was used in all patients. All surgeries were performed between 2012 and 2014 in a single‐stage procedure using the LIT‐TR.

#### 2.1.2 Study B: LIT‐TP versus LIT‐TR

Study B compared LIT‐TP with LIT‐TR for inserting BAHIs.^5^, ^6^ The test group (25 patients with 25 implants) was implanted with a wide Ponto implant and underwent single‐stage surgery in 2014 using the LIT‐TP.^3^ Abutment length was based on skin thickness. The control group consisted of the last 25 patients (25 implants) who already had participated in the test group of study A.

### 2.2 Patients, follow‐up, and outcome measures

Patients who had completed the 3‐year follow‐up of study A or B were invited to participate in a single visit 5 years after implantation. Outcomes comprised implant stability, implant survival, soft tissue status, skin height and revision surgery. Additionally, skin sensitivity around the abutment and subjective numbness were assessed in patients who had participated in study B. Implant stability was measured by means of the ISQ using resonance frequency analysis and a SmartPeg 55 (Osstell AB, Göteborg, Sweden). The IPS scale was used in addition to the Holgers‐scale to assess soft tissue status.^9^ A Holgers‐score ≥2, or an IPS‐score indicating treatment, were considered an adverse skin reaction. Skin height was evaluated relative to the abutment.

### 2.3 Data analyses

Data analyses were executed by independent external biostatisticians (Statistiska Konsultgruppen, Göteborg, Sweden). All tests were performed using SAS v9.4 (Cary, NC), were two‐tailed and conducted at .05 significance level.

### 2.4 Ethical considerations

This clinical investigation was performed in accordance with the declaration of Helsinki (Washington 2002, ISO 14155), Good Clinical Practice (International Conference on Harmonisation Good Clinical Practice) and was approved by the local ethical committee. Informed consent was obtained for all patients.

## 3 RESULTS

### 3.1 Patients

In study A, 48 patients (51 implants) attended the 5‐year follow‐up visit (84%). The test group consisted of 33 patients (34 implants) and the control group of 17 patients (17 implants). In study B, 39 patients completed the 5‐year follow‐up (78%). See Figure S1. Between groups, no significant differences were observed at baseline in both studies (Table 1).

**TABLE 1:** Baseline characteristics of all patients included in study A and B

	Study A		Study B	
Variable	4.5‐mm‐wide implant *(test) n = 39*	3.75‐mm‐wide implant *(control) n = 20*	LIT‐TP *(test) n = 25*	LIT‐TR *(control) n = 25*
Gender, *n* (%)				
Male	15 (38.5)	9 (45.0)	15 (60.0)	10 (40.0)
Female	24 (61.5)	11 (55.0)	10 (40.0)	15 (60.0)
Age in years, mean (SD)	53.7 (12.0)	53.0 (16.4)	51.5 (13.4)	53.9 (12.2)
Aetiology, *n* (%)				
Acquired cond./mixed	26 (66.7)^a^	16 (80.0)	21 (84.0)	18 (72.0)
Congenital conductive	1 (2.6)^a^	1 (5.0)	1 (4.0)	0 (0.0)
Single‐sided deafness	13 (33.3)^a^	3 (15.0)	3 (12.0)	7 (28.0)
Implants, *n* (%)				
Single implant	36 (91.2)	18 (90.0)	25 (100)	25 (100)
Two identical implants	1 (2.6)	0 (0.0)	0 (0.0)	0 (0.0)
Two different implants	2 (5.1)	2 (10.0)	0 (0.0)	0 (0.0)

Abbreviations: LIT‐TP, linear incision technique with soft tissue preservation; LIT‐TP, linear incision technique with soft tissue reduction.

^a^ One bilaterally implanted patient had two different indications for a bone anchored hearing device.

### 3.2 Implant and abutment survival

See Table 2. Between the 3‐year and 5‐year visit, no additional implant losses occurred. One abutment was removed in a patient with a 4.5‐mm‐wide implant (study A) because of cochlear implantation. Between the 4.5‐mm and 3.75‐mm‐wide implant, no significant differences in implant survival (97.4% versus 95.0%) or abutment survival (94.8% versus 95.0%) were observed. Furthermore, between patients who underwent implantation with a 4.5‐mm‐wide implant using either the LIT‐TP or LIT‐TR, implant survival (96% versus 100%) and abutment survival (92% versus 92%) were comparable.

**TABLE 2:** Outcome measures at the 5‐year visit

	Study A		Study B	
Variable	4.5‐mm‐wide implant *(test)*	3.75‐mm‐wide implant *(control)*	LIT‐TP *(test)*	LIT‐TR *(control)*
Implant survival rate	38 (97.4%)	19 (95.0%)	24 (96%)	25 (100%)
Abutment survival rate	37 (94.8%)	19 (95.0%)	23 (92%)	23 (92%)
Adverse skin reaction rate (Holgers)	0 (0.0%)	1 (5.9%)	0 (0.0%)	0 (0.0%)
Adverse skin reaction rate (IPS)	1 (3.0%)	3 (17.6%)	0 (0.0%)	0 (0.0%)
Skin sensitivity	—	—	100%	100%

Abbreviations: LIT‐TP, linear incision technique with soft tissue preservation; LIT‐TP, linear incision technique with soft tissue reduction.

### 3.3 Soft tissue tolerability and complications

See Table 2. Figure S2 shows Holgers‐scores across visits, as well as the maximum Holgers‐score per implant. Over the 5‐year follow‐up period, adverse Holgers‐scores (Holgers ≥ 2) were observed in 15.2% of the 4.5‐mm‐wide implants and in 23.5% of the 3.75‐mm‐wide implants (*p* = .72) for patients operated with LIT‐TR.

At 5‐year follow‐up, adverse IPS‐scores were observed in 3.0% of the 4.5‐mm‐wide implants and 17.6% of the 3.75‐mm‐wide implants (*p* = .22). Adverse Holgers‐scores were reported in 0.0% of the 4.5‐mm‐wide implants and 5.9% of the 3.75‐mm‐wide implants (*p* = .68). See Table 3.

**TABLE 3:** Specified soft tissue reactions according to the IPS‐scale at the 5‐year visit

	Study A		Study B	
Variable	4.5‐mm‐wide implant *(test) n = 33*	3.75‐mm‐wide implant *(control) n = 17*	LIT‐TP *(test) n = 20*	LIT‐TR *(control) n = 19*
Inflammation‐score, *n* (%)				
Skin integrity	0 (0.0)	0 (0.0)	0 (0.0)	0 (0.0)
Erythema	4 (12.1)	5 (29.4)	1 (5.0)	2 (10.5)
Edema	0 (0.0)	0 (0.0)	0 (0.0)	0 (0.0)
Granulation	1 (3.0)	2 (11.8)	0 (0.0)	0 (0.0)
Pain‐score, *n* (%)				
None	32 (97.0)	14 (82.4)	20 (100.0)	19 (100.0)
Present, <6 weeks	1 (3.0)	2 (11.8)	0 (0.0)	0 (0.0)
Present, >6 weeks	0 (0.0)	1 (5.9)	0 (0.0)	0 (0.0)
Skin height‐score, *n* (%)				
Normal	32 (97.0)	14 (82.4)	20 (100.0)	19 (100.0)
Increased	1 (3.0)	3 (17.6)	0 (0.0)	0 (0.0)
Above abutment rim	0 (0.0)	0 (0.0)	0 (0.0)	0 (0.0)
Total IPS‐score, *n* (%)				
I0 P0 S0	28 (84.8)	10 (58.8)	19 (95.0)	17 (89.5)
I0 P2 S0	0 (0.0)	1 (5.9)	0 (0.0)	0 (0.0)
I1 P0 S0	3 (9.1)	2 (11.8)	1 (5.0)	2 (10.5)
I1 P0 S1	1 (3.0)	2 (11.8)	0 (0.0)	0 (0.0)
I1 P1 S0	1 (3.0)	1 (5.9)	0 (0.0)	0 (0.0)
I2 P1 S1	0 (0.0)	1 (5.9)	0 (0.0)	0 (0.0)

Abbreviations: LIT‐TP, linear incision technique with soft tissue preservation; LIT‐TP, linear incision technique with soft tissue reduction.

In the LIT‐TP versus LIT‐TR group, adverse Holgers‐scores were observed on at least one occasion over the 5‐year period in 30.0% and 10.5% of the implants, respectively (*p* = .27). Figure S3 shows the proportion of adverse Holgers‐scores during the 5‐year follow‐up for the LIT‐TP and LIT‐TR group. No adverse Holgers nor IPS scores were observed at 5‐year.

Between the 3‐ and 5‐year visit, one patient underwent revision surgery, as mentioned earlier.

At 5‐year, skin height did not significantly differ between the 4.5‐mm and 3.75‐mm‐wide implants, nor between LIT‐TP and LIT‐TR group. For both groups, distance between skin and shoulder of the abutment decreased over time resulting in an increased skin height (65.4% and 71.8% respectively), which never led to problems with sound processor coupling. In addition, no correlation between skin height and adverse Holgers‐scores was observed.

### 3.4 Skin sensitivity

At the 5‐year follow‐up, total sensitivity was comparable between LIT‐TP and LIT‐TR with a median total sensitivity of 100% (range 83.3–100) for LIT‐TP and 100% (range 75–100) for LIT‐TR (*p* = .82), and a mean total sensitivity of 96.7% (SD 5.7) for LIT‐TP and 96.3% (SD 7.1) for LIT‐TR (*p* = .82). At the 5‐year visit, none of the patients in either group experienced subjective numbness.

## 4 DISCUSSION

### 4.1 Main findings and clinical applicability

The current study presents data of a 5‐year single follow‐up visit of patients who underwent BAHI implantation in two previously conducted clinical studies.^5^, ^6^, ^7^ This study provides the reader with high‐quality data and a complete overview of long‐term results.

High 5‐year implant and abutment survival rates were observed. No significant differences in implant and abutment survival were observed between subgroups. However, this study had insufficient power to detect differences in survival. Although the number of adverse skin reactions differed widely between subgroups, no significant differences were found at 5‐year follow‐up or during 5‐year follow‐up. Skin sensitivity was also similar between subgroups during and at 5‐year follow‐up. The difference in adverse skin reaction rate might be explained by the limited number of patients per subgroup.

Following LIT‐TR and LIT‐TP, comparable skin height was observed at 5 years follow‐up. No correlation between skin height and adverse skin reactions was found. Therefore, on long‐term, the use of a wide percutaneous implant and the tissue preservation technique leads to high implant survival and low percentages of adverse skin reactions.

As result of evolvement of implants and surgical techniques, decision‐making regarding implant type and surgical procedure has gotten more complicated over the years. Our results have confirmed the excellent survival rates of both implant types, with higher resonance frequency properties for the 4.5‐mm‐wide implant. Nonetheless, no previous studies were able to show better implant survival for the 4.5‐mm‐wide implant, presumably due to inadequate sample sizes.^8^ Further research supporting larger sample sizes should be conducted to answer this question. When comparing surgical techniques, the LIT‐TP is considered to have shorter surgery time and better cosmetic outcomes compared with the LIT‐TR.^6^ No differences in adverse skin reactions nor implant survival were observed in this study. With the current state of knowledge, we believe both implants are safe on long‐term with excellent implant survival rates. Because of shorter surgery time, less invasive character and comparable clinical outcomes, LIT‐TP is currently preferred over LIT‐TR.

### 4.2 Strengths and limitations

The current study is the first long‐term follow‐up study evaluating both surgical technique and implant design. Data quality is high, as only one outcome measure was missing in one patient. However, with nine patients lost to follow‐up, selection bias might have occurred.

### 4.3 Comparison with other studies

To the best of our knowledge, this is the first study to compare 5‐year outcomes of the 3.75‐mm‐wide and the 4.5‐mm‐wide Ponto implants. Yet, 5‐year outcomes of another 4.5‐mm‐wide implant type were evaluated in a multicentre study.^10^ In contrast to our study, the 4.5‐mm‐wide implant investigated by den Besten et al., has shown to be superior in terms of adverse Holgers‐scores. This result could be explained by use of two different abutment types, whereas in the current study similar abutments were used for both implants. Only one long‐term comparative study on LIT‐TP and LIT‐TR has been published.^4^ Reznitsky et al. followed patients for 4 years (LIT‐TP) or for 5 years (LIT‐TR). They reported high‐implant survival. Adverse skin reactions were in line with our findings.

## 5 CONCLUSION

Independent of implant design and surgical technique, high implant and abutment survival rates were observed for patients implanted with both the 3.75‐mm‐wide implant and the 4.5‐mm‐wide implant. Implant survival rate was 95% for the 3.75‐mm‐wide implant and ranged between 96% (LIT‐TP) and 100% (LIT‐TR) for the 4.5‐mm‐wide implant. At 5 years, adverse skin reactions occurred in a minority of implants and did not significantly differ between groups. It can therefore be concluded that both 4.5‐mm‐wide implant and the LIT‐TP procedure are safe in the long‐term.

## AUTHOR CONTRIBUTIONS

IK EM MH designed the work; MV CC IK acquired and analysed data; all authors drafted, revised and approved the manuscript; all authors agree to be accountable for all aspects of the work.

## FUNDING INFORMATION

Oticon Medical AB (Askim, Sweden) provided financial support for this study.

The authors report financial support to the authors' institution (Radboudumc) for conducting clinical studies from Oticon Medical AB (Askim, Sweden) and from Cochlear Bone Anchored Solutions AB (Mölnlycke, Sweden), also outside the submitted work.

## CONFLICT OF INTEREST

The authors declare that they have no other conflict of interest.

### PEER REVIEW

The peer review history for this article is available at https://publons.com/publon/10.1111/coa.13974.

## Supporting information


**Appendix S1** Supporting Information

**Figure S1** Flowchart demonstrating the number of patients participating in the study over time. Reasons for withdrawal‐included lost‐to‐follow‐up, deceased patient, elective removal of abutment, and patient's decision to discontinue trial.

**Figure S2** **A** Box‐and‐Whisker plots of ISQ‐low and ‐high values per implant for study A, comparing a 4.5‐mm and 3.75‐mm‐wide implant. Analyses performed on 6 mm abutments exclusively. **B**: Box‐and‐Whisker plots of ISQ‐low and ‐high values per implant for study B, comparing LIT‐TP and LIT‐TR. Abutment size varied from 6 to 12 mm in the LIT‐TP group, whereas only 6 mm abutments were used in the LIT‐TR group. LIT‐TP indicates linear incision technique with soft tissue preservation; LIT‐TR, linear incision technique with soft tissue reduction; ISQ, implant stability quotient.

**Figure S3** Holgers‐grade across visits (A) and maximum Holgers grade (B) per implant for study A, comparing a 4.5‐mm‐wide and 3.75‐mm‐wide implant. Holgers‐grade across visits (C) and maximum Holgers grade (D) per implant for study B, comparing the linear incision technique with soft tissue preservation and linear incision technique with tissue reduction

**Figure S4** Proportion of adverse skin reactions measured with the Holgers score between the linear incision technique with soft tissue preservation and linear incision technique with tissue reduction at 6 months, 1, 2, 3, and 5 years follow‐up

## Data Availability

The data that support the findings of this study are available from the corresponding author upon reasonable request.
