# Supplementary material for: Prospective 5 year outcomes of different implant designs and surgical techniques in 68 patients with bone anchored hearing implants
Source: Clin Otolaryngol. 2022 Sep 17;48(1):65–9. doi: 10.1111/coa.13974 (PMC10087793; doi:10.1111/coa.13974)
Supplement: Supplementary file 1 — Appendix S1 Supporting Information [file COA-48-65-s003.docx]

**SUPPLEMENTAL DATA AND FIGURES**

Data analyses
Data-analyses of the 5-year follow-up data were separately conducted for patients who had originally participated in study A and for patients who had participated in study B. Analyses on implant stability and implant survival were performed on all patients. In case of premature withdrawal from the original studies, or patients not participating in the 5-year follow-up, all collected data to the point of withdrawal were included in the analyses on implant stability and implant survival. All other outcome measures were analyzed for the patients participating in the 5-year follow-up. In case of missing variables in the 5-year follow-up population, the last-observation-carried-forward method was used.

For statistical analyses, nonparametric statistics were used. Groups were compared using the Mann–Whitney *U* test for continuous variables, the Mantel–Haenszel χ^2^ test for ordered categorical variables, the Fisher’s exact test for dichotomous variables, and the χ^2^ test for non-ordered categorical variables. Repeated measures analyses were done for changes over time. For analyses over time, the Wilcoxon signed rank test was used for continuous variables and the Sign test was used for ordered categorical variables and dichotomous variables. To compare implant survival between groups, the Logrank survival test was used.

Results

**Implant Stability Quotient**
The ISQ-low and -high are displayed in Supplemental Figure 4. For study A, the inter-group differences for both the mean AUC of ISQ-low and high were statistically significant at the 5-year follow-up (*p*=.0028 and *p*=.029, respectively). Between 2 and 3 years after surgery, a significant decrease in ISQ-low and -high had been observed for the 4.5-mm-wide implants^9^. At the 5-year visit, a further decrease in ISQ-low (1.8; *p*=.033) was seen in this group, compared with the 3-year visit. For the 3.75-mm-wide implants, a slight, non-significant, increase in ISQ is observed since the 2-year visit.

A comparison of ISQ-values between LIT-TP and LIT-TR (study B) resulted in a significantly higher mean AUC of ISQ-low and ISQ-high for LIT-TR, as we would expect as a result of the differences in abutment length (65.1 vs 60.8, respectively; *p*=.0008 and 66.5 vs 62.2, respectively; *p*=.0004). The increase in ISQ-low from surgery until the 5-year visit was significantly greater in the LIT-TP group compared to the LIT-TR group (7.85 vs 1.63, respectively; *p*=.0013). For ISQ-high, a lower, but also statistically significant, increase was observed (7.60 vs 2.53, respectively; *p*=.0027).

**FIGURES**

**Supplemental figure 1** Flowchart demonstrating the number of patients participating in the study over time. Reasons for withdrawal included lost-to-follow-up, deceased patient, elective removal of abutment, and patient’s decision to discontinue trial.

**Supplemental figure 2** **A** Box-and-Whisker plots of ISQ-low and -high values per implant for study A, comparing a 4.5-mm and 3.75-mm-wide implant. Analyses performed on 6 mm abutments exclusively. **B**: Box-and-Whisker plots of ISQ-low and -high values per implant for study B, comparing LIT-TP and LIT-TR. Abutment size varied from 6–12 mm in the LIT-TP group, whereas only 6 mm abutments were used in the LIT-TR group. LIT-TP indicates linear incision technique with soft tissue preservation; LIT-TR, linear incision technique with soft tissue reduction; ISQ, implant stability quotient.

**Supplemental Figure 3** Holgers-grade across visits (A) and maximum Holgers grade (B) per implant for study A, comparing a 4.5-mm-wide and 3.75-mm-wide implant. Holgers-grade across visits (C) and maximum Holgers grade (D) per implant for study B, comparing the linear incision technique with soft tissue preservation and linear incision technique with tissue reduction

**Supplemental Figure 4** Proportion of adverse skin reactions measured with the Holgers score between the linear incision technique with soft tissue preservation and linear incision technique with tissue reduction at 6 months, 1 year, 2 years, 3 years, and 5 years follow-up
