# Supplementary figures and images for: Prospective 5 year outcomes of different implant designs and surgical techniques in 68 patients with bone anchored hearing implants
Source: Clin Otolaryngol. 2022 Sep 17;48(1):65–9. doi: 10.1111/coa.13974 (PMC10087793; doi:10.1111/coa.13974)

Start of study

3-year follow-up

5-year follow-up

Study A  
Test

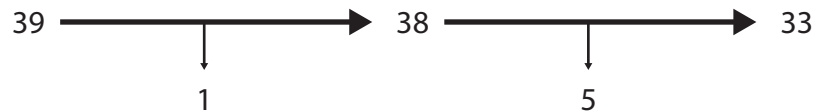

Control

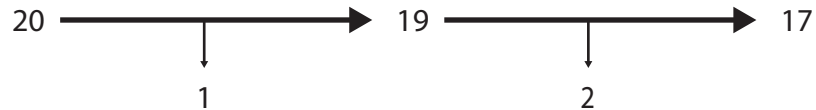

Study B  
Test

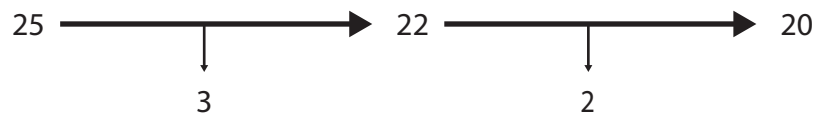

Control

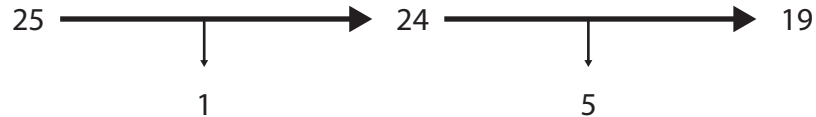

Supplement: Supplementary file 2 — Figure S1 Flowchart demonstrating the number of patients participating in the study over time. Reasons for withdrawal‐included lost‐to‐follow‐up, deceased patient, elective removal of abutment, and patient's decision to discontinue trial. [file COA-48-65-s005.pdf]

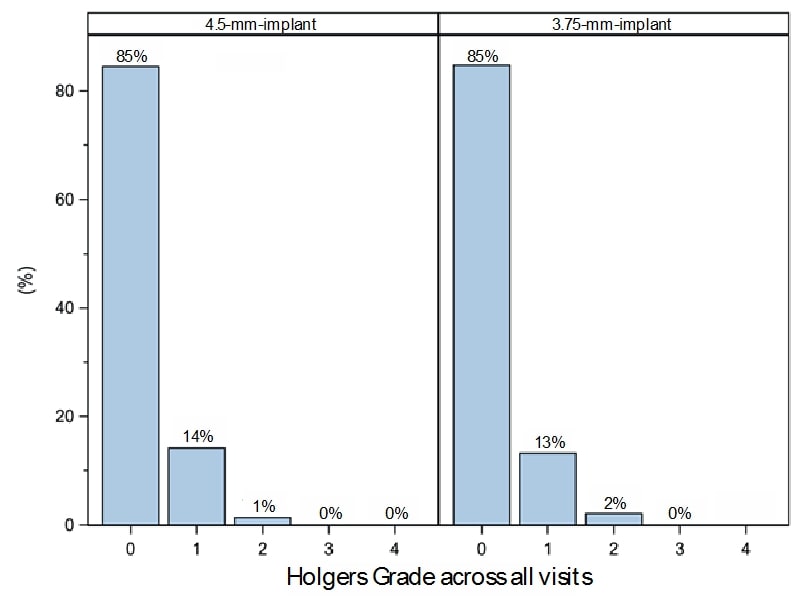

Supplement: Supplementary file 3 — Figure S2 A Box‐and‐Whisker plots of ISQ‐low and ‐high values per implant for study A, comparing a 4.5‐mm and 3.75‐mm‐wide implant. Analyses performed on 6 mm abutments exclusively. B: Box‐and‐Whisker plots of ISQ‐low and ‐high values per implant for study B, comparing LIT‐TP and LIT‐TR. Abutment size varied from 6 to 12 mm in the LIT‐TP group, whereas only 6 mm abutments were used in the LIT‐TR group. LIT‐TP indicates linear incision technique with soft tissue preservation; LIT‐TR, linear incision technique with soft tissue reduction; ISQ, implant stability quotient. [file COA-48-65-s001.zip › COA_13974_Supplementary figure 2A.jpg]

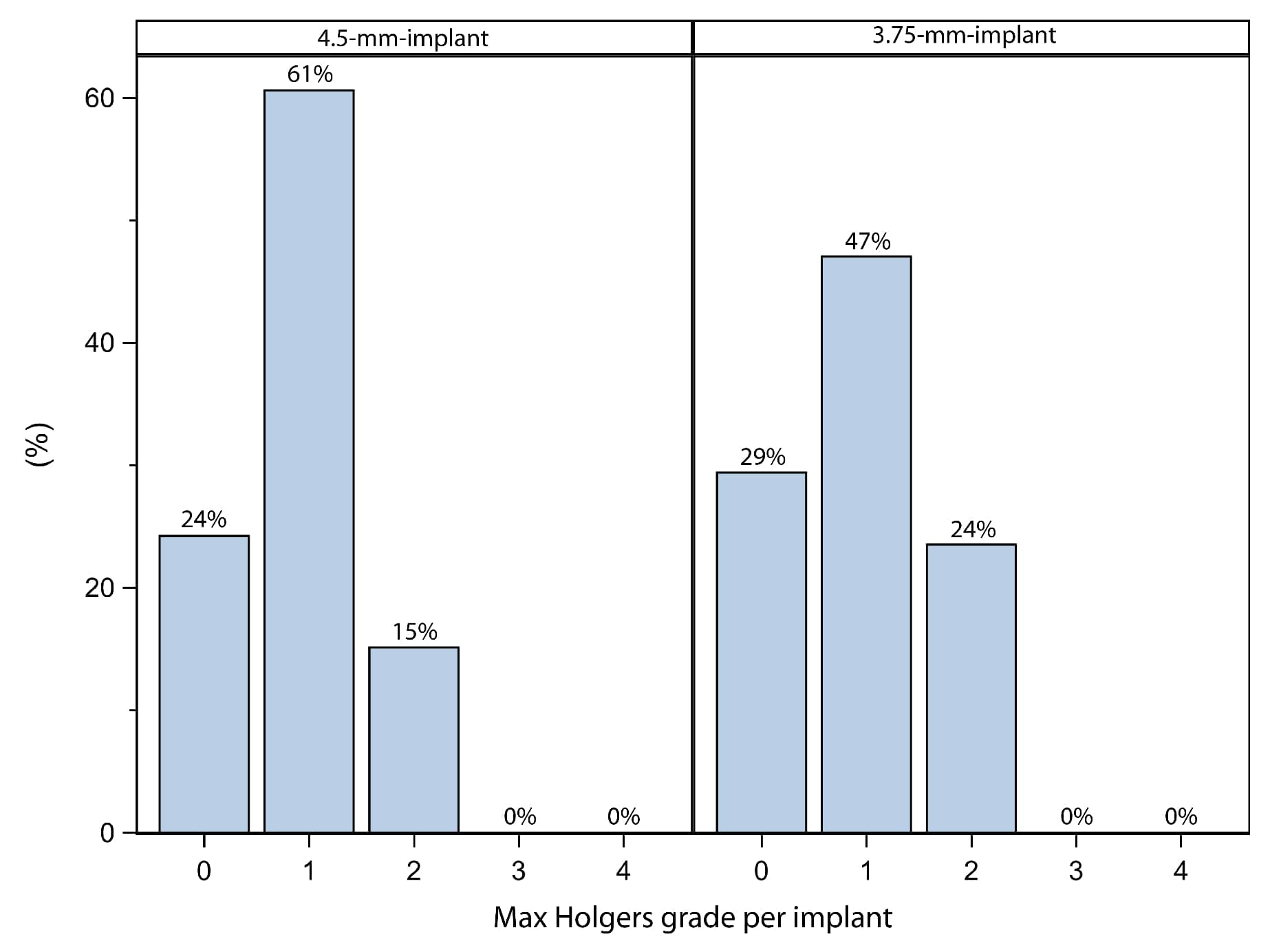

Supplement: Supplementary file 3 — Figure S2 A Box‐and‐Whisker plots of ISQ‐low and ‐high values per implant for study A, comparing a 4.5‐mm and 3.75‐mm‐wide implant. Analyses performed on 6 mm abutments exclusively. B: Box‐and‐Whisker plots of ISQ‐low and ‐high values per implant for study B, comparing LIT‐TP and LIT‐TR. Abutment size varied from 6 to 12 mm in the LIT‐TP group, whereas only 6 mm abutments were used in the LIT‐TR group. LIT‐TP indicates linear incision technique with soft tissue preservation; LIT‐TR, linear incision technique with soft tissue reduction; ISQ, implant stability quotient. [file COA-48-65-s001.zip › COA_13974_Supplementary figure 2B.jpg]

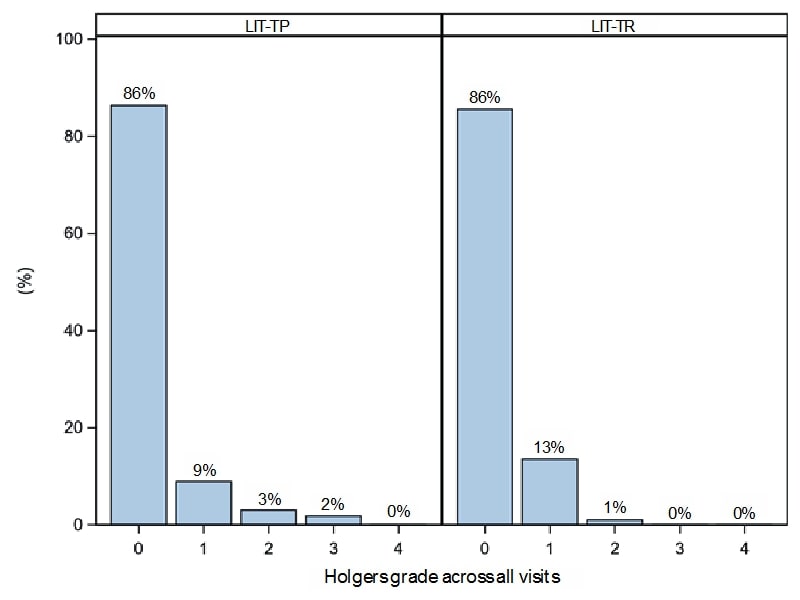

Supplement: Supplementary file 3 — Figure S2 A Box‐and‐Whisker plots of ISQ‐low and ‐high values per implant for study A, comparing a 4.5‐mm and 3.75‐mm‐wide implant. Analyses performed on 6 mm abutments exclusively. B: Box‐and‐Whisker plots of ISQ‐low and ‐high values per implant for study B, comparing LIT‐TP and LIT‐TR. Abutment size varied from 6 to 12 mm in the LIT‐TP group, whereas only 6 mm abutments were used in the LIT‐TR group. LIT‐TP indicates linear incision technique with soft tissue preservation; LIT‐TR, linear incision technique with soft tissue reduction; ISQ, implant stability quotient. [file COA-48-65-s001.zip › COA_13974_Supplementary figure 2C.jpg]

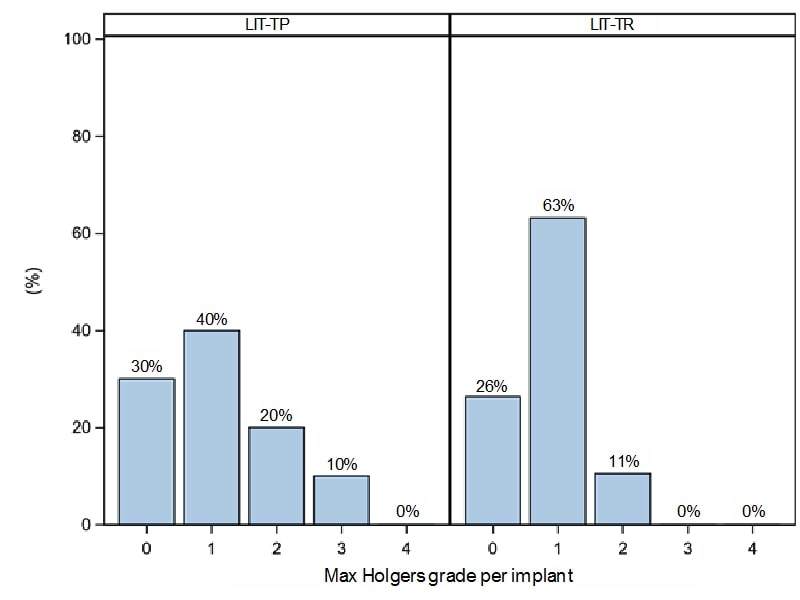

Supplement: Supplementary file 3 — Figure S2 A Box‐and‐Whisker plots of ISQ‐low and ‐high values per implant for study A, comparing a 4.5‐mm and 3.75‐mm‐wide implant. Analyses performed on 6 mm abutments exclusively. B: Box‐and‐Whisker plots of ISQ‐low and ‐high values per implant for study B, comparing LIT‐TP and LIT‐TR. Abutment size varied from 6 to 12 mm in the LIT‐TP group, whereas only 6 mm abutments were used in the LIT‐TR group. LIT‐TP indicates linear incision technique with soft tissue preservation; LIT‐TR, linear incision technique with soft tissue reduction; ISQ, implant stability quotient. [file COA-48-65-s001.zip › COA_13974_Supplementary figure 2D.jpg]

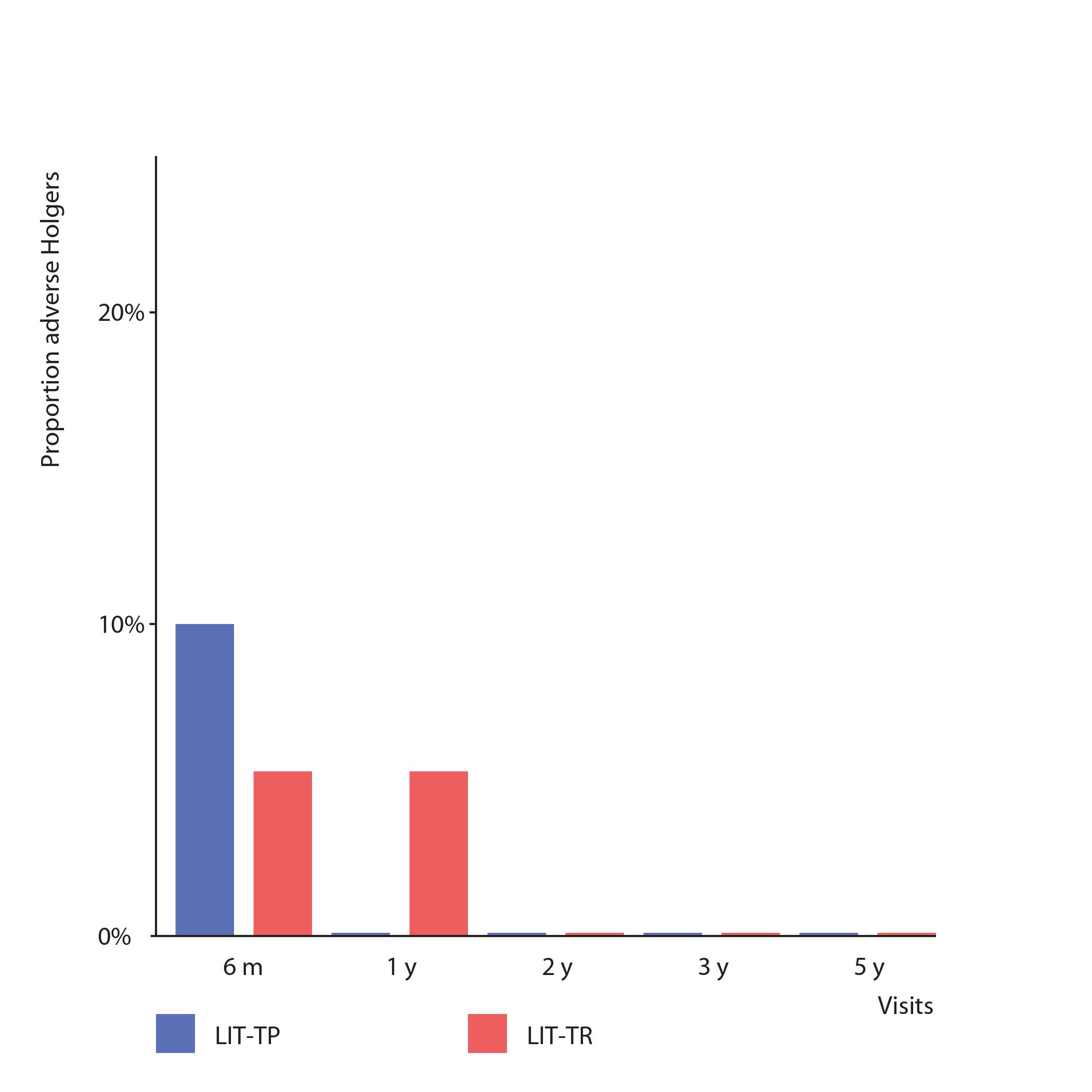

Supplement: Supplementary file 4 — Figure S3 Holgers‐grade across visits (A) and maximum Holgers grade (B) per implant for study A, comparing a 4.5‐mm‐wide and 3.75‐mm‐wide implant. Holgers‐grade across visits (C) and maximum Holgers grade (D) per implant for study B, comparing the linear incision technique with soft tissue preservation and linear incision technique with tissue reduction [file COA-48-65-s004.jpg]

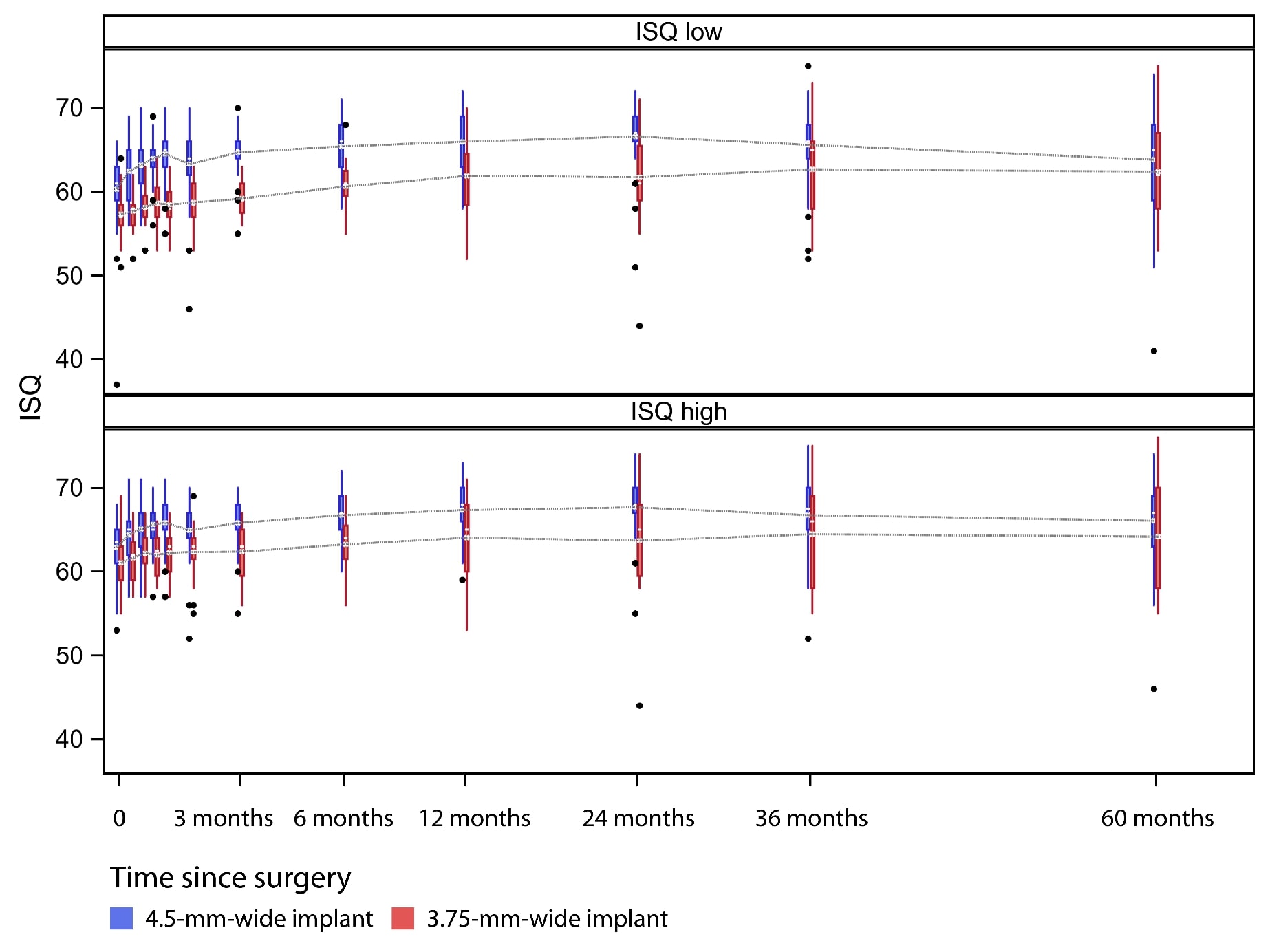

Supplement: Supplementary file 5 — Figure S4 Proportion of adverse skin reactions measured with the Holgers score between the linear incision technique with soft tissue preservation and linear incision technique with tissue reduction at 6 months, 1, 2, 3, and 5 years follow‐up [file COA-48-65-s002.zip › COA_13974_Supplementary figure 4A.jpg]

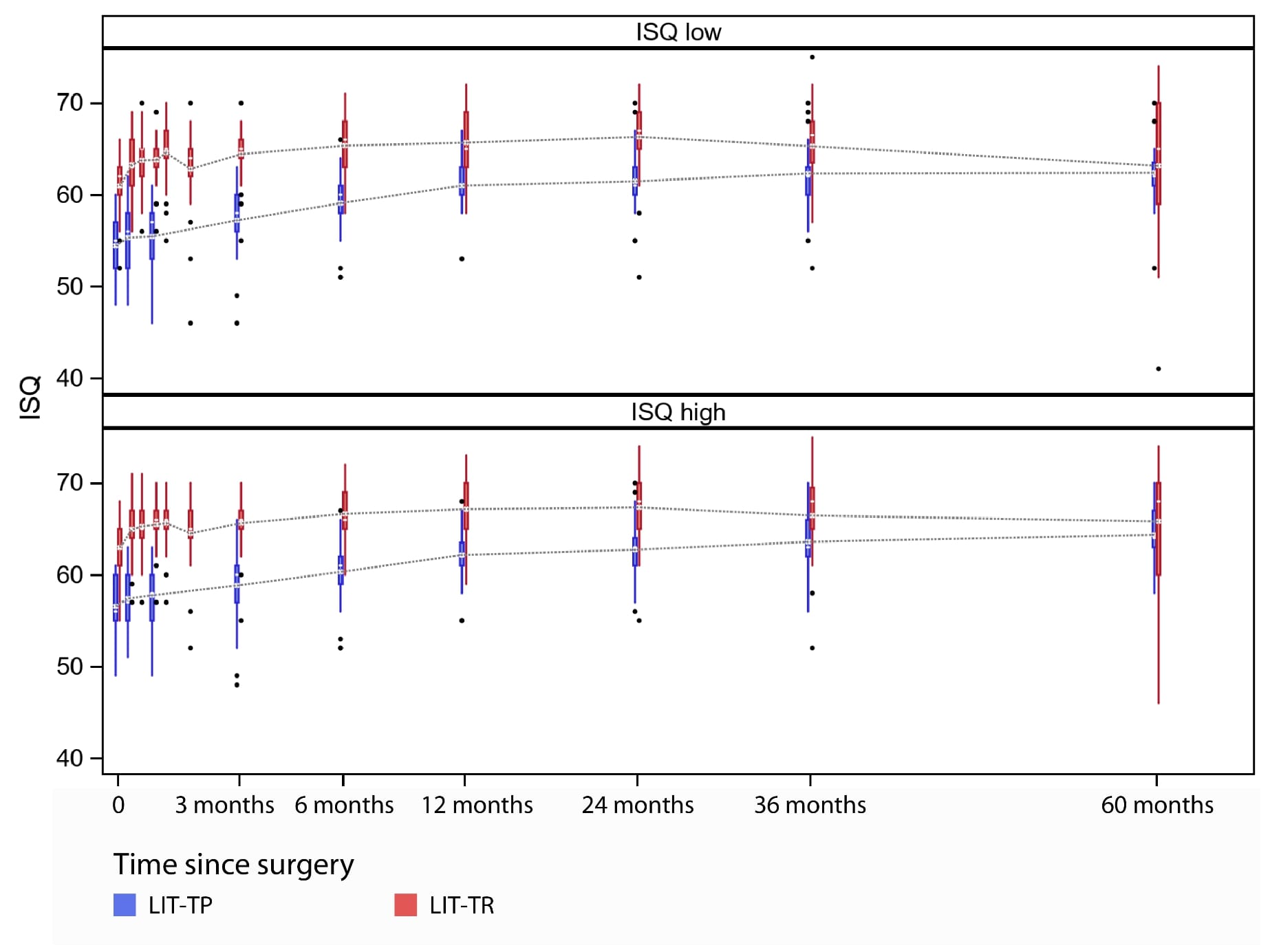

Supplement: Supplementary file 5 — Figure S4 Proportion of adverse skin reactions measured with the Holgers score between the linear incision technique with soft tissue preservation and linear incision technique with tissue reduction at 6 months, 1, 2, 3, and 5 years follow‐up [file COA-48-65-s002.zip › COA_13974_Supplementary figure 4B.jpg]
